# Supplementary material for: Concordance rate of radiologists and a commercialized deep-learning solution for chest X-ray: Real-world experience with a multicenter health screening cohort
Source: PLoS One. 2022 Feb 24;17(2):e0264383. doi: 10.1371/journal.pone.0264383 (PMC8870572; doi:10.1371/journal.pone.0264383)
Supplement: Attachment — Submitted filename: Response to Reviewers_211108.docx [file pone.0264383.s003.docx]

19-November-2021

Dear Alfredo Vellido
Academic Editor, PLOS ONE

Thank you very much for the opportunity to revise our original article entitled “Concordance rate of radiologists and a commercialized deep-learning solution for chest X-ray: Real-world experience with a multicenter health screening cohort (PONE-D-21-19659).” After carefully reading the reviewer’ and editor’s comments, we have tried to improve the quality and legibility of the manuscript according to the points raised.

In the revised version, changes are indicated by highlighting. Individual points (E-editor’s comments and R-#1 = point 1 made by reviewer) are indicated in red.

**Editor’s comment**

PONE-D-21-19659

Concordance rate of radiologists and a commercialized deep-learning solution for chest X-ray: Real-world experience with a multicenter health screening cohort

PLOS ONE

Dear Dr. Jin,

Thank you for submitting your manuscript to PLOS ONE. After careful consideration, we feel that it has merit but does not fully meet PLOS ONE’s publication criteria as it currently stands. Therefore, we invite you to submit a revised version of the manuscript that addresses the points raised during the review process.

Please submit your revised manuscript by Dec 18 2021 11:59PM. If you will need more time than this to complete your revisions, please reply to this message or contact the journal office at [plosone@plos.org](mailto:plosone@plos.org). When you're ready to submit your revision, log on to <https://www.editorialmanager.com/pone/> and select the 'Submissions Needing Revision' folder to locate your manuscript file.

Please include the following items when submitting your revised manuscript:

- A rebuttal letter that responds to each point raised by the academic editor and reviewer(s). You should upload this letter as a separate file labeled 'Response to Reviewers'.
- A marked-up copy of your manuscript that highlights changes made to the original version. You should upload this as a separate file labeled 'Revised Manuscript with Track Changes'.
- An unmarked version of your revised paper without tracked changes. You should upload this as a separate file labeled 'Manuscript'.

If you would like to make changes to your financial disclosure, please include your updated statement in your cover letter. Guidelines for resubmitting your figure files are available below the reviewer comments at the end of this letter.

If applicable, we recommend that you deposit your laboratory protocols in [protocols.io](http://protocols.io/) to enhance the reproducibility of your results. Protocols.io assigns your protocol its own identifier (DOI) so that it can be cited independently in the future. For instructions see: <https://journals.plos.org/plosone/s/submission-guidelines#loc-laboratory-protocols>. Additionally, PLOS ONE offers an option for publishing peer-reviewed Lab Protocol articles, which describe protocols hosted on [protocols.io](http://protocols.io/). Read more information on sharing protocols at <https://plos.org/protocols?utm_medium=editorial-email&utm_source=authorletters&utm_campaign=protocols>.

We look forward to receiving your revised manuscript.

Kind regards,

Alfredo Vellido
Academic Editor
PLOS ONE

Journal Requirements:

When submitting your revision, we need you to address these additional requirements.

1. Please ensure that your manuscript meets PLOS ONE's style requirements, including those for file naming. The PLOS ONE style templates can be found at

<https://journals.plos.org/plosone/s/file?id=wjVg/PLOSOne_formatting_sample_main_body.pdf> and <https://journals.plos.org/plosone/s/file?id=ba62/PLOSOne_formatting_sample_title_authors_affiliations.pdf>.

 🡪 Yes, we did.

2. Thank you for stating the following in the Competing Interests/Financial Disclosure* (delete as necessary) section:

“KNJ has received research grant funding from Lunit Inc., outside the present study. This does not alter our adherence to PLOS ONE policies on sharing data and materials. Other authors have no potential conflicts of interest to disclose”

We note that one or more of the authors are employed by a commercial company: name of commercial company.

🡪 No authors are employed by a commercial company.

a. Please provide an amended Funding Statement declaring this commercial affiliation, as well as a statement regarding the Role of Funders in your study. If the funding organization did not play a role in the study design, data collection and analysis, decision to publish, or preparation of the manuscript and only provided financial support in the form of authors' salaries and/or research materials, please review your statements relating to the author contributions, and ensure you have specifically and accurately indicated the role(s) that these authors had in your study. You can update author roles in the Author Contributions section of the online submission form.

Please also include the following statement within your amended Funding Statement.

“The funder provided support in the form of salaries for authors [insert relevant initials], but did not have any additional role in the study design, data collection and analysis, decision to publish, or preparation of the manuscript. The specific roles of these authors are articulated in the ‘author contributions’ section.”

If your commercial affiliation did play a role in your study, please state and explain this role within your updated Funding Statement.

🡪 In cover letter, I add the amended funding statement as follows;

**Updated Funding Statement:**

This work was supported by a grant from the Korea Health Industry Development Institute to YJC (Grant number: HI19C0847). The funder had no role in study design, data collection and analysis, decision to publish, or preparation of the manuscript.

b. Please also provide an updated Competing Interests Statement declaring this commercial affiliation along with any other relevant declarations relating to employment, consultancy, patents, products in development, or marketed products, etc.

Within your Competing Interests Statement, please confirm that this commercial affiliation does not alter your adherence to all PLOS ONE policies on sharing data and materials by including the following statement: "This does not alter our adherence to  PLOS ONE policies on sharing data and materials.” (as detailed online in our guide for authors <http://journals.plos.org/plosone/s/competing-interests>) . If this adherence statement is not accurate and  there are restrictions on sharing of data and/or materials, please state these. Please note that we cannot proceed with consideration of your article until this information has been declared.

Please include both an updated Funding Statement and Competing Interests Statement in your cover letter. We will change the online submission form on your behalf.

🡪 In cover letter, I add the COI statement as follows;

KNJ has received research grant funding from Lunit Inc. for activities not related to the

present article. This does not alter our adherence to PLOS ONE policies on sharing data and materials. Other authors have no potential conflicts of interest to disclose.

3. In your Data Availability statement, you have not specified where the minimal data set underlying the results described in your manuscript can be found. PLOS defines a study's minimal data set as the underlying data used to reach the conclusions drawn in the manuscript and any additional data required to replicate the reported study findings in their entirety. All PLOS journals require that the minimal data set be made fully available. For more information about our data policy, please see <http://journals.plos.org/plosone/s/data-availability>.

"Upon re-submitting your revised manuscript, please upload your study’s minimal underlying data set as either Supporting Information files or to a stable, public repository and include the relevant URLs, DOIs, or accession numbers within your revised cover letter. For a list of acceptable repositories, please see <http://journals.plos.org/plosone/s/data-availability#loc-recommended-repositories>. Any potentially identifying patient information must be fully anonymized.

Important: If there are ethical or legal restrictions to sharing your data publicly, please explain these restrictions in detail. Please see our guidelines for more information on what we consider unacceptable restrictions to publicly sharing data: <http://journals.plos.org/plosone/s/data-availability#loc-unacceptable-data-access-restrictions>. Note that it is not acceptable for the authors to be the sole named individuals responsible for ensuring data access.

We will update your Data Availability statement to reflect the information you provide in your cover letter.

🡪 In cover letter, I add the Data Availability statement as follows;

All relevant data are within the manuscript and its Supporting Information files.

Reviewers' comments:

Reviewer's Responses to Questions

**Comments to the Author**

1. Is the manuscript technically sound, and do the data support the conclusions?

The manuscript must describe a technically sound piece of scientific research with data that supports the conclusions. Experiments must have been conducted rigorously, with appropriate controls, replication, and sample sizes. The conclusions must be drawn appropriately based on the data presented.

Reviewer #1: Yes

Reviewer #2: Partly

2. Has the statistical analysis been performed appropriately and rigorously?

Reviewer #1: Yes

Reviewer #2: Yes

3. Have the authors made all data underlying the findings in their manuscript fully available?

The [PLOS Data policy](http://www.plosone.org/static/policies.action#sharing) requires authors to make all data underlying the findings described in their manuscript fully available without restriction, with rare exception (please refer to the Data Availability Statement in the manuscript PDF file). The data should be provided as part of the manuscript or its supporting information, or deposited to a public repository. For example, in addition to summary statistics, the data points behind means, medians and variance measures should be available. If there are restrictions on publicly sharing data—e.g. participant privacy or use of data from a third party—those must be specified.

Reviewer #1: Yes

Reviewer #2: No

🡪 Thank you for your comment. We added supporting information file.

4. Is the manuscript presented in an intelligible fashion and written in standard English?

PLOS ONE does not copyedit accepted manuscripts, so the language in submitted articles must be clear, correct, and unambiguous. Any typographical or grammatical errors should be corrected at revision, so please note any specific errors here.

Reviewer #1: Yes

Reviewer #2: Yes

5. Review Comments to the Author

Please use the space provided to explain your answers to the questions above. You may also include additional comments for the author, including concerns about dual publication, research ethics, or publication ethics. (Please upload your review as an attachment if it exceeds 20,000 characters)

Reviewer #1: Summary:
Artificial intelligence (AI) has great potential to fundamentally alter healthcare delivery; however, to data most clinical evaluations commonly concern about artificial elements and often ultimately neglect the human-in-the-loop AI. Success of AI in diagnostic imaging has fueled a growing debate surrounding whether the comprehensive diagnostic interpretive skillsets of radiologist can be replicated by algorithms. This is a nice study timely answer the important question about what’s the role of AI in real-world clinical practice. From this study, we understand that human and AI should be more partners other than competitors. Results of the study revealed high agreement between human and AI in a real-world multicenter health screening cohort.
🡪 Thank you for your comments.

Limitation:
1) There is no follow-up outcome or absence of experts’ consensus reference to evaluate the diagnostic performance of AI. Even the authors argued that they is aimed to evaluate application of AI in a real-world situation rather than focus to the diagnostic accuracy, the accuracy is still first important for a new AI tool in clinical practice. If a new tool is just consistent with experts while did not bring new additional benefit or improvement (such as accuracy, sensitivity or time-saving) to radiologists, we do not know why we use it. So, how is useful of this AI tool to radiologists’ interpretation is yet unknown for us. Please clarify.

🡪 Thank you for your comments. Many of published data shows comparable diagnostic accuracy of AI. We wanted to show the clinical value for the AI application at real-world setting. In real-world setting, diagnostic accuracy is hard to measure because of 1) hard to get additional consensus reading for too many X-rays 2) if we include cases underwent CT (regarded gold standard test), it cannot avoid selection bias and cannot reflect the real world situation (in real world, only less than 10% underwent CT). 3) FU period was not that enough to show the prognosis of patients.

Nevertheless, we wanted to show how the main users (radiologist) confidently accept the result of AI tool for real-world setting. In addition, we also evaluated the consumption time for CXR reading before and after the adaptation of AI.

Finally, we added System Usability Scale as indicated Reviewer #2, and some discussion.

2) AI were compared with only three radiologists, which is not representative in a real world clinical setting. The interpretation from only three readers may had high variances, potentially impacting the results of study.

🡪 Thank you for your comments. We added this point at limitation section.

Reviewer #2: 1. The manuscript shares 18% similarity with the previously published study which was not cited in the reference:
<https://doi.org/10.1371/journal.pone.0246472>
🡪 Thank you for your comments. We added the paper in the reference.

2. Proper citation to the previous work is required and clarification must be made whether the data used in this study overlap with the published one.
🡪 Thank you for your comments for our mistake. We added the paper in the reference (as indicated R2-#1). However, the study cohort is totally different and the data used in this study doesn’t overlap at all with data in the previous work.

3. In "Introduction", line 4 - 5 from the bottom, "no study has evaluated the extent to which radiologists accept the Lunit results in real-world clinical practice". The gap identified here was not addressed in the current study. Different tools must be used for this (e.g., System Usability Scale, UTAUT etc.).
🡪 Thank you for your comments. As you indicated, we evaluated how the radiologists confidently accept the result of AI tool for real-world setting, not the general usability of AI. We added the System Usability Scale surveillance for radiologists, radiologist residents and non-radiology physician who were interested in our study.

4. According to the PLOS Data policy, it requires authors to make all data underlying the findings described in their manuscript fully available without restriction, with rare exception (please refer to the Data Availability Statement in the manuscript PDF file). The data should be provided as part of the manuscript or its supporting information, or deposited to a public repository. For example, in addition to summary statistics, the data points behind means, medians and variance measures should be available. If there are restrictions on publicly sharing data—e.g. participant privacy or use of data from a third party—those must be specified. No link to the raw data were provided in the manuscript.
🡪 Thank you for your comment. We added supporting information files.

5. High concordance result of Lunit is indirectly related to the accuracy of the performance as published in the previous manuscript. It is unclear how this study can contribute more insight to the performance of Lunit. The advantages of the incorporation of Lunit in the existing workflow is also not clearly specified (e.g., can it be used in health screening centers without the assistance of radiologist? Or perhaps as training tools for future or inexperienced radiologist?).

🡪 Thank you for your comment. We added the description at discussion section.

6. PLOS authors have the option to publish the peer review history of their article ([what does this mean?](https://journals.plos.org/plosone/s/editorial-and-peer-review-process#loc-peer-review-history)). If published, this will include your full peer review and any attached files.


If you choose “no”, your identity will remain anonymous but your review may still be made public.


**Do you want your identity to be public for this peer review?** For information about this choice, including consent withdrawal, please see our [Privacy Policy](https://www.plos.org/privacy-policy).

Reviewer #1: **Yes:** Yu-Dong Zhang

Reviewer #2: No

[NOTE: If reviewer comments were submitted as an attachment file, they will be attached to this email and accessible via the submission site. Please log into your account, locate the manuscript record, and check for the action link "View Attachments". If this link does not appear, there are no attachment files.]

While revising your submission, please upload your figure files to the Preflight Analysis and Conversion Engine (PACE) digital diagnostic tool, <https://pacev2.apexcovantage.com/>. PACE helps ensure that figures meet PLOS requirements. To use PACE, you must first register as a user. Registration is free. Then, login and navigate to the UPLOAD tab, where you will find detailed instructions on how to use the tool. If you encounter any issues or have any questions when using PACE, please email PLOS at [figures@plos.org](mailto:figures@plos.org). Please note that Supporting Information files do not need this step.

Thank you again for giving us great honor to resubmit our manuscript to this prestigious journal. We have done our best to respond to all points indicated by the reviewers. We hope you find the revised manuscript acceptable for publication in PLOS ONE.

The authors confirm the manuscript has not been published previously and that it will not be submitted for publication elsewhere and the authors have no conflict of interest to declare.

With best regards,

From Authors

Uploaded:

-Revised and highlighted versions of the revised manuscript (‘Manuscript’ file and

‘Revised Manuscript with Track Changes’ file)

-Rebuttal letter (‘Responses to Reviewers’ file)
