# Supplementary material for: Concordance rate of radiologists and a commercialized deep-learning solution for chest X-ray: Real-world experience with a multicenter health screening cohort
Source: PLoS One. 2022 Feb 24;17(2):e0264383. doi: 10.1371/journal.pone.0264383 (PMC8870572; doi:10.1371/journal.pone.0264383)
Supplement: Attachment — Submitted filename: Response to Reviewers_220118.docx [file pone.0264383.s004.docx]

18-January-2022

Dear Alfredo Vellido
Academic Editor, PLOS ONE

Happy New Year!

Thank you very much for the opportunity to revise our original article entitled “Concordance rate of radiologists and a commercialized deep-learning solution for chest X-ray: Real-world experience with a multicenter health screening cohort (PONE-D-21-19659).” After carefully reading the reviewer’ and editor’s comments, we have tried to improve the quality and legibility of the manuscript according to the points raised.

In the revised version, changes are indicated by highlighting. Individual points (E-editor’s comments and R-#1 = point 1 made by reviewer) are indicated in red.

PONE-D-21-19659R1

Concordance rate of radiologists and a commercialized deep-learning solution for chest X-ray: Real-world experience with a multicenter health screening cohort

PLOS ONE

Dear Dr. Jin,

Thank you for submitting your manuscript to PLOS ONE. After careful consideration, we feel that it has merit but does not fully meet PLOS ONE’s publication criteria as it currently stands. Therefore, we invite you to submit a revised version of the manuscript that addresses the points raised during the review process.

Please submit your revised manuscript by Feb 18 2022 11:59PM. If you will need more time than this to complete your revisions, please reply to this message or contact the journal office at [plosone@plos.org](mailto:plosone@plos.org). When you're ready to submit your revision, log on to <https://www.editorialmanager.com/pone/> and select the 'Submissions Needing Revision' folder to locate your manuscript file.

Please include the following items when submitting your revised manuscript:

- A rebuttal letter that responds to each point raised by the academic editor and reviewer(s). You should upload this letter as a separate file labeled 'Response to Reviewers'.
- A marked-up copy of your manuscript that highlights changes made to the original version. You should upload this as a separate file labeled 'Revised Manuscript with Track Changes'.
- An unmarked version of your revised paper without tracked changes. You should upload this as a separate file labeled 'Manuscript'.

If you would like to make changes to your financial disclosure, please include your updated statement in your cover letter. Guidelines for resubmitting your figure files are available below the reviewer comments at the end of this letter.

If applicable, we recommend that you deposit your laboratory protocols in [protocols.io](http://protocols.io/) to enhance the reproducibility of your results. Protocols.io assigns your protocol its own identifier (DOI) so that it can be cited independently in the future. For instructions see: <https://journals.plos.org/plosone/s/submission-guidelines#loc-laboratory-protocols>. Additionally, PLOS ONE offers an option for publishing peer-reviewed Lab Protocol articles, which describe protocols hosted on [protocols.io](http://protocols.io/). Read more information on sharing protocols at <https://plos.org/protocols?utm_medium=editorial-email&utm_source=authorletters&utm_campaign=protocols>.

We look forward to receiving your revised manuscript.

Kind regards,

Alfredo Vellido
Academic Editor
PLOS ONE

[Note: HTML markup is below. Please do not edit.]

Reviewers' comments:

Reviewer's Responses to Questions

**Comments to the Author**

1. If the authors have adequately addressed your comments raised in a previous round of review and you feel that this manuscript is now acceptable for publication, you may indicate that here to bypass the “Comments to the Author” section, enter your conflict of interest statement in the “Confidential to Editor” section, and submit your "Accept" recommendation.

Reviewer #1: (No Response)

Reviewer #2: (No Response)

2. Is the manuscript technically sound, and do the data support the conclusions?

The manuscript must describe a technically sound piece of scientific research with data that supports the conclusions. Experiments must have been conducted rigorously, with appropriate controls, replication, and sample sizes. The conclusions must be drawn appropriately based on the data presented.

Reviewer #1: Partly

Reviewer #2: Yes

3. Has the statistical analysis been performed appropriately and rigorously?

Reviewer #1: Yes

Reviewer #2: Yes

4. Have the authors made all data underlying the findings in their manuscript fully available?

The [PLOS Data policy](http://www.plosone.org/static/policies.action#sharing) requires authors to make all data underlying the findings described in their manuscript fully available without restriction, with rare exception (please refer to the Data Availability Statement in the manuscript PDF file). The data should be provided as part of the manuscript or its supporting information, or deposited to a public repository. For example, in addition to summary statistics, the data points behind means, medians and variance measures should be available. If there are restrictions on publicly sharing data—e.g. participant privacy or use of data from a third party—those must be specified.

Reviewer #1: Yes

Reviewer #2: No

5. Is the manuscript presented in an intelligible fashion and written in standard English?

PLOS ONE does not copyedit accepted manuscripts, so the language in submitted articles must be clear, correct, and unambiguous. Any typographical or grammatical errors should be corrected at revision, so please note any specific errors here.

Reviewer #1: Yes

Reviewer #2: Yes

6. Review Comments to the Author

Please use the space provided to explain your answers to the questions above. You may also include additional comments for the author, including concerns about dual publication, research ethics, or publication ethics. (Please upload your review as an attachment if it exceeds 20,000 characters)

Reviewer #1: In my initial comments on LIMITATION, I mentioned that only three radiologists were recruited for review, which is not clinically representative. While the response is not satisfactory by just adding a clarification of limitation. The more appropriate anwer is to take a more head to head comparsion betwen different readers, e.g., a team of juniors vs a team of seniors.

🡪 Thank you for your comments. We totally agree with you. As you indicated, we evaluate the concordance of AI results with the CXR reading made by only three radiologists, which might not be representative results. However, it also reflected the real clinical environment that only several radiologists were solely in charge of CXR for health screening centers. Actually, there was only one staff radiologist in charge of CXR interpretation in three health care centers.

As a retrospective study, the original clinical radiology reports was made by three board-certified radiologists from three health screening centers (one per institution; C.S.Y., K.R.H., K.S., with 11, 7, and 20 years of experience in radiology, respectively) and we retrospectively analyzed the concordance of AI results and the original radiology reports.

Reviewer #2: - Missing data in S2 Appendix, additional data are required for concordance/discordance rate calculation.

🡪 We checked the data in S2 Appendix. AI result and concordance is not available for control group (group 2, without AI application group)

- Typographic errors:
1) In "Abstract", misspelling of questionnaire "Finally, we evaluated... questionair...".
2) In "System Usability Scale", misspelling of total, "A toal of 24 radiologists and physicians..."
- Please check other typo errors throughout the manuscript prior to the final submission.

🡪 Thank you very much. We amended the typo throughout the manuscript.

7. PLOS authors have the option to publish the peer review history of their article ([what does this mean?](https://journals.plos.org/plosone/s/editorial-and-peer-review-process#loc-peer-review-history)). If published, this will include your full peer review and any attached files.


If you choose “no”, your identity will remain anonymous but your review may still be made public.


**Do you want your identity to be public for this peer review?** For information about this choice, including consent withdrawal, please see our [Privacy Policy](https://www.plos.org/privacy-policy).

Reviewer #1: No

Reviewer #2: No

[NOTE: If reviewer comments were submitted as an attachment file, they will be attached to this email and accessible via the submission site. Please log into your account, locate the manuscript record, and check for the action link "View Attachments". If this link does not appear, there are no attachment files.]

While revising your submission, please upload your figure files to the Preflight Analysis and Conversion Engine (PACE) digital diagnostic tool, <https://pacev2.apexcovantage.com/>. PACE helps ensure that figures meet PLOS requirements. To use PACE, you must first register as a user. Registration is free. Then, login and navigate to the UPLOAD tab, where you will find detailed instructions on how to use the tool. If you encounter any issues or have any questions when using PACE, please email PLOS at [figures@plos.org](mailto:figures@plos.org). Please note that Supporting Information files do not need this step.

Thank you again for giving us great honor to resubmit our manuscript to this prestigious journal. We have done our best to respond to all points indicated by the reviewers. We hope you find the revised manuscript acceptable for publication in PLOS ONE.

The authors confirm the manuscript has not been published previously and that it will not be submitted for publication elsewhere and the authors have no conflict of interest to declare.

With best regards,

From Authors

Uploaded:

-Revised and highlighted versions of the revised manuscript (‘Manuscript’ file and

‘Revised Manuscript with Track Changes’ file)

-Rebuttal letter (‘Responses to Reviewers’ file)
